# The Impact of Diversity Inclusion Practices in the Workplace Context: The Effect of Inclusive Leadership

**DOI:** 10.3390/ejihpe15070121

**Published:** 2025-07-02

**Authors:** Silvia Platania, Claudio Maggio, Marcello Boccadamo

**Affiliations:** Department of Educational Science, Section of Psychology, University of Catania, 95124 Catania, Italy; claudio.maggio@phd.unict.it (C.M.); marcello.boccadamo@outlook.com (M.B.)

**Keywords:** diversity, inclusive leadership, job dissatisfaction

## Abstract

The present study explores the predictive role of organisational identification in shaping both positive and negative employee responses and the potential mediating influence of diversity climate and inclusive leadership within this relationship. Specifically, it examines how employees’ organisational identification influences their perceptions of diversity climate and inclusive leadership and how these factors, in turn, mediate their responses to organisational dissatisfaction. This study involved 307 participants from the Italian public administration who were administered a questionnaire to measure organisational identification, inclusive leadership (Inclusive Leadership Scale), the diversity climate within the organisation, and behaviours according to the EVLN model. The results indicate a direct effect of organisational identification on both the positive (Voice and Loyalty) and negative (Exit and Neglect) responses of the EVLN model. Organisational identification has a positive effect on the diversity climate. Moreover, the diversity climate mediates the relationship between organisational identification and loyalty, while inclusive leadership mediates the relationship between organisational identification and both disengagement and the willingness to address issues. These findings underscore the central role of organisational identification in shaping employees’ behavioural responses to dissatisfaction by influencing their perceptions of diversity climate and inclusive leadership. This highlights the importance of strengthening organisational identification to foster constructive behaviours and mitigate negative responses in diverse and inclusive work contexts.

## 1. Introduction

Identification with the organisation is a key element in human resource management and organisational behaviour, as it influences multiple aspects of working life. Organisational identification (OID) refers to the extent to which employees define themselves in terms of their membership in an organisation, internalising its values and goals as part of their self-concept. It represents a psychological bond between the individual and the organisation.

OID does not develop independently; rather, it is strongly shaped by management practices and the organisational climate. Two key elements that foster the strengthening of OID are inclusive leadership and a diversity-oriented climate.

Inclusive leadership, characterised by active listening, valuing opinions, and recognising individual contributions, fosters a sense of identification and strengthens OID (Randel et al., 2018). When leaders cultivate a fair and participatory work environment, employees are more likely to perceive the organisation as part of their identity. Diversity climate is a further determining factor for organisational identification (McKay et al., 2009). Effective diversity management ensures that all employees, regardless of their individual differences, feel valued and included, thereby fostering a positive bond with the organisation (Mor Barak et al., 2016).

Diversity climate, defined as the degree to which a firm advocates fair human resource policies and socially integrates underrepresented employees (McKay et al., 2008), is grounded in Social Identity Theory. This theory posits that individuals categorise themselves and others into social groups and, based on these categorisations, make decisions favouring the in-group at the expense of the outgroup (Tajfel & Turner, 1986).

Rooted in Social Identity Theory (Tajfel & Turner, 1986), the concept of diversity climate refers to employees’ shared perceptions regarding the extent to which an organisation promotes and values diversity and inclusion. From this perspective, a positive diversity climate serves as a contextual cue that affirms the value of diverse social identities within the workplace, thereby strengthening employees’ organisational identification. This theoretical linkage aligns with the Interactional Model of Cultural Diversity (IMCD), (Cox, 1994), which posits that employees’ responses to diversity are shaped by the dynamic interaction between structural elements (e.g., policies, leadership practices) and individuals’ lived experiences. Integrating diversity climate into the IMCD framework allows for a more comprehensive understanding of how organisational structures influence identity-related processes and behavioural outcomes (Hofhuis et al., 2012).

The IMCD views diversity not as a fixed characteristic but as a process shaped by ongoing interactions between individuals and the organisational context. Within this framework, management practices are designed to actively promote equality, inclusion, and integration through open communication, equitable policies, and inclusive leadership. These practices foster a climate in which all employees feel valued and fairly treated, thereby reinforcing organisational identification and pro-social behaviour. In turn, these practices positively influence employees’ subjective experiences and performance (Cox, 1994). The underlying rationale is that such policies enable workers to perceive the organisation as a cohesive social group to which they belong, encouraging behaviours that support the in-group—namely, the organisation itself.

Given the significant variability of the diversity climate construct, which has led several authors to criticise its inconsistency (Holmes et al., 2021), some scholars have structured it into three key components: intentionality, programming, and praxis (Cachat-Rosset et al., 2019).

Intentionality includes declarative elements, such as management statements explicitly endorsing diversity. Programming encompasses operational elements, including policy implementation, corporate initiatives, and the establishment of standards aimed at improving worker integration. Praxis refers to the practical dimension, specifically the execution of planned initiatives and the behaviours exhibited by members of the organisation (Platania et al., 2022a, 2022b). Several studies have highlighted how organisational identification represents a central construct in determining employee behaviour, particularly in situations of dissatisfaction or conflict (Ashforth & Mael, 1989). The process of internalising organisational identity profoundly influences the way employees respond to problematic workplace situations and is closely linked to the responses outlined in the EVLN model (Exit, Voice, Loyalty, Neglect), originally proposed by Hirschman (1970) and later expanded by Rusbult et al. (1988).

In particular, organisational identification emerges as a significant predictor of the response strategies adopted by employees. A strong sense of identification tends to reduce the likelihood of enacting exit or neglect strategies, instead orienting behaviour towards active participation (voice) or organisational loyalty. Employees who identify with their organisation are less inclined to leave in times of difficulty, as they perceive it as part of their social self (Van Dick et al., 2004). Similarly, they are more likely to respond with constructive engagement, seeking to positively influence the working environment through dialogue and active involvement (Van Dyne & LePine, 1998). Loyalty, understood as the patient and trusting expectation of improvement, represents another typical response among those who experience a strong sense of identification (Mael & Ashforth, 1995). Conversely, low levels of organisational identification are often associated with neglectful behaviours, apathy, and a gradual withdrawal from one’s professional role (Blader & Tyler, 2009).

Based on these theoretical considerations, the general aim of this study is to explore the impact of diversity climate and inclusive leadership on organisational dynamics and employees’ subjective experiences within the Italian public administration. In particular, this study examines the mediating role of these factors in the relationship between organisational identification and employees’ responses, both positive and negative. The analysis aims to provide valuable insights for the development of organisational strategies to foster a more inclusive and cohesive work environment.

## 2. Theoretical Background

### 2.1. Inclusive Leadership: Fostering Diversity, Equity, and Identification in Organisations

In the context of increasing workplace diversity, and with particular attention to the concept of diversity climate—especially its three core components—the role of leadership emerges as a critical factor. Given that inclusive leadership remains a relatively recent and underexplored construct, this study aims to examine how it influences both positive and negative employee behaviours. Leadership plays a fundamental role in shaping the organisation’s approach to diversity and in fostering a supportive environment. It contributes to building a clear, cohesive vision that is easily recognisable to employees, thereby promoting a shared sense of identity and belonging (Mor Barak et al., 2016).

Inclusive leadership (Korkmaz et al., 2022) is a management approach that places inclusion, respect, and the appreciation of diversity at the heart of organisational practice. This leadership style is grounded in the belief that an inclusive environment not only enhances employee well-being but also drives creativity, innovation, and productivity.

Inclusive leadership thus operates on two levels: directly, by influencing organisational policies related to work organisation and evaluation, and indirectly, by shaping employee experiences through these policies. These dynamics are framed within the broader concepts of procedural justice (Lind & Tyler, 2013) and distributive justice (Jafino et al., 2021).

Organisational justice is typically conceptualised through three dimensions: procedural, distributive, and interactional justice. Procedural justice suggests that when leaders allocate rewards and sanctions based on transparent, consistently applied rules—perceived as legitimate by employees—individuals are more likely to feel respected within the organisation, thereby fostering stronger identification with the group. Distributive justice relates to employees’ perceptions of fairness in the distribution of outcomes, which shapes their sense of equity and inclusion. Interactional justice refers to perceived fairness in the quality of interpersonal treatment and communication from organisational representatives.

Inclusive leadership is intrinsically linked to all three forms of justice. By promoting fair procedures, the equitable distribution of resources, and respectful, open interactions, inclusive leaders reinforce employees’ sense of belonging and organisational identification.

Conversely, when leadership is perceived as non-inclusive, employees may find it difficult to identify with the organisation—particularly if they feel that they, or their social group, are being devalued. A diminished sense of organisational identification (OID) can, in turn, increase the likelihood of psychological withdrawal and disengagement (Kreiner & Ashforth, 2004).

### 2.2. EVLN Model

The EVLN model (Exit, Voice, Loyalty, and Neglect), originally developed by Hirschman (1970), provides a comprehensive framework for analysing how employees respond to dissatisfaction or perceived problems within an organisation. The model outlines a general taxonomy of behavioural responses, encompassing both constructive and destructive reactions. Its flexibility allows for the integration of various psychological and organisational antecedents, making it particularly suitable for examining how factors such as organisational identification, inclusive leadership, and diversity climate influence workplace behaviour (Hirschman, 1970).

The EVLN model is grounded in the notion that all organisations inevitably experience periods of qualitative decline due to internal or external factors. As such, monitoring general organisational quality alone may be insufficient. Instead, the model identifies four specific behavioural responses—Exit, Voice, Loyalty, and Neglect—that can serve as early indicators of organisational dysfunction and inform corrective action (Farrell, 1983). These responses are positioned along two intersecting dimensions: Active vs. Passive and Constructive vs. Destructive. Exit refers to behaviours through which employees withdraw from the organisation. This may involve inter-organisational turnover (leaving the organisation entirely) or intra-organisational turnover (moving to a different role within the same organisation) (Jackofsky & Peters, 1983). The latter is particularly noteworthy, as it may go undetected through traditional turnover metrics but can reveal deeper structural issues. Exit is classified as Active and Destructive, as it reflects the belief that problems are unlikely to be resolved. Voice involves actively attempting to address and resolve organisational issues by communicating them to a superior. Rather than withdrawing, employees who engage in Voice seek to restore organisational functioning to previous levels of effectiveness (Hirschman, 1970; Steers & Mowday, 1981). This response is viewed as Active and Constructive, signalling both awareness of problems and a willingness to contribute to their resolution. Loyalty is characterised by continued support for the organisation in the face of problems, without overt action to address them (Rusbult et al., 1988). It is often associated with employee silence, which can take either a positive form (adhesion silence, motivated by trust and alignment with the organisation) or a negative form (ejection silence, driven by fear, resignation, or submission) (Sabino et al., 2019). Loyalty is typically considered Passive and Constructive, though some authors argue that it may become destructive when it prevents the expression of critical concerns. Neglect includes passive behaviours that result in performance decline, whether intentional or not. Examples include absenteeism, procrastination, careless errors, or time-wasting (Rusbult et al., 1988). Although not explicitly aimed at harming the organisation, these behaviours undermine performance and are classified as Passive and Destructive. These four behavioural responses show distinct patterns of correlation with job satisfaction (Li et al., 2024). Specifically, job satisfaction is positively associated with both Voice and Loyalty, while it is negatively associated with Exit and Neglect. Given that job satisfaction is itself correlated with organisational identification (Karanika-Murray et al., 2015), enhancing employees’ sense of identification with the organisation may help to increase constructive responses such as Voice and Loyalty, while simultaneously reducing destructive responses such as Exit and Neglect.

### 2.3. The Effect of Inclusive Leadership and the Responses from Employees

The theoretical and practical interplay between diversity, inclusive leadership, and the EVLN model (Exit, Voice, Loyalty, and Neglect) is essential for understanding how the dynamics of inclusion and diversity shape employee behaviour and the organisational climate. Each component of this triad interacts with the others, implying that an organisation’s approach to diversity and inclusive leadership can directly influence how employees respond to dissatisfaction and organisational challenges.

Diversity within an organisation refers to the presence of differences among its members—such as ethnicity, gender, sexual orientation, cultural background, and other dimensions. Inclusive leadership plays a critical role in cultivating a work environment in which all individuals, regardless of their personal characteristics, feel respected, valued, and integrated (Shore et al., 2018).

An inclusive leader not only embraces diversity as a value but also leverages it as a strategic asset to enhance innovation, creativity, and overall performance. This leadership style fosters a psychologically safe environment that encourages employees to express themselves freely. More specifically, inclusive leadership influences key psychological processes by promoting perceptions of fairness, strengthening organisational identification, and enhancing empowerment. These mechanisms help reduce negative behaviours (Exit, Neglect) and reinforce positive responses (Voice, Loyalty), thereby explaining its mediating role.

From a theoretical perspective, diversity and inclusive leadership function as antecedents of employee behaviour, as described by the EVLN model. Organisations that support diversity and practice inclusive leadership are more likely to reduce the incidence of Exit and Neglect while promoting Voice and Loyalty (Rusbult et al., 1988).

From a practical standpoint, companies that adopt diversity initiatives and foster inclusive leadership can reduce employee turnover (Exit), increase engagement (Voice), and build a committed, motivated workforce (Loyalty). In doing so, they also prevent detrimental behaviours (Neglect) that compromise work quality and organisational performance (Avery et al., 2008).

Ultimately, the connection between diversity, inclusive leadership, and the EVLN model illustrates how organisations that value and promote inclusion can positively shape employee behaviour. An inclusive leader not only champions diversity but also guides the organisation toward constructive responses, preventing dysfunctional reactions that may hinder its effectiveness.

Diversity in an organisation refers to the presence of differences among its members, which may include ethnicity, gender, sexual orientation, cultural background, and many other dimensions. Inclusive leadership plays a key role in fostering an environment where every individual, regardless of their personal characteristics, feels valued and integrated (Shore et al., 2018).

An inclusive leader recognises and promotes diversity not only as a value but as an asset that can strengthen innovation, creativity, and business performance. The inclusive approach entails fostering a safe and respectful environment in which all employees are encouraged to express themselves openly and authentically. In particular, the mediating role of inclusive leadership can be better understood by considering its influence on key psychological processes. Specifically, inclusive leadership fosters perceptions of fairness, strengthens employees’ sense of identification, and promotes empowerment. These factors contribute to reducing negative behaviours (Exit, Neglect) and enhancing positive ones (Voice, Loyalty), thereby explaining the mechanism underlying its mediating function.

Theoretically, diversity and inclusive leadership act as factors that influence employee behaviour, as outlined in the EVLN model. An organisation that promotes diversity and adopts inclusive leadership reduces the likelihood of Exit and Neglect, while encouraging more positive responses such as Voice and Loyalty (Rusbult et al., 1988).

Practically, companies that implement diversity policies and foster inclusive leadership can reduce turnover rates (Exit), improve employee engagement (Voice), and encourage a motivated and committed workforce (Loyalty), thus preventing harmful behaviours (Neglect) that could undermine work quality and productivity (Avery et al., 2008).

The connection between diversity, inclusive leadership, and the EVLN model demonstrates how an organisational environment that values diversity and promotes inclusive practices can positively influence employee responses, leading to a healthier and more productive work climate. An inclusive leader not only promotes diversity but also guides the organisation towards constructive and productive behaviours, preventing negative reactions that could harm the organisation.

### 2.4. Aim of the Research

The aim of this study is to conduct an exploratory analysis within the Italian public administration, to investigate the predictive effect that organisational identification may have on employees’ responses, both positive and negative, and whether diversity climate and inclusive leadership can also have a mediating effect in this relationship.

**Figure 1:**
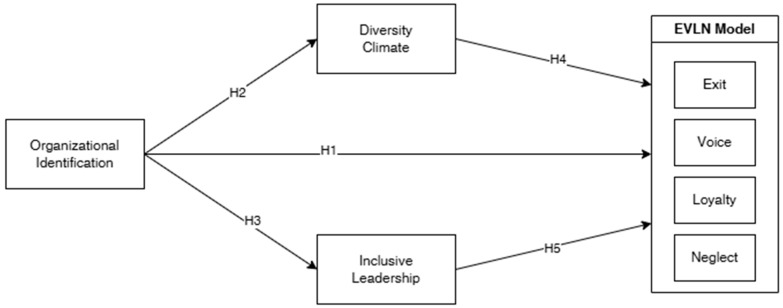
Theoretical model.

In particular, the following hypotheses will be tested (see Figure 1):

**H1.**  *Organisational Identification has a direct positive effect on the positive responses of the EVLN model (Voice and Loyalty).*

**H2.**  *Organisational Identification has a direct positive effect on perceptions of Diversity Climate.*

**H3.**  *Organisational Identification has a direct positive effect on perceptions of Inclusive Leadership.*

**H4.**  *Diversity Climate mediates the relationship between Organisational Identification and the EVLN model variables (Voice and Loyalty).*

**H5.**  *Inclusive Leadership mediates the relationship between Organisational Identification and the EVLN model variables (Voice and Loyalty).*

## 3. Method

### 3.1. Participants and Procedures

This study involved a total of 307 participants, primarily from Southern Italy (78.4%) and predominantly employed in the public administration sector (82.6%). The sample included 181 male participants (59%), 122 female participants (39.7%), and 4 participants identifying as gender fluid (1.3%). The average length of service was 11.1 years (SD = 3.9).

Most participants worked in various sectors of the Italian public administration, including healthcare (39%), schools (16.1%), and universities (27.5%). Consequently, the study employed a convenience sampling method.

Participants were recruited through the collaboration of human resources departments, which contacted potential respondents via email. Each participant received a link to an online questionnaire hosted on Google Forms, along with an introductory letter outlining the study’s objectives. Before beginning the survey, participants provided informed consent electronically.

The completion of the survey required approximately 15 min. Participation was entirely voluntary, and individuals could discontinue the survey at any point without any obligation to proceed. The study adhered to the principles of the Declaration of Helsinki and received approval from the Internal Ethics Review Board of the Department of Educational Sciences (Section of Psychology) of the University of Catania (Prot. No. Ierb-Edunict-2020.010.13/5).

### 3.2. Measures

#### 3.2.1. The Organisational Identification Scale (OIS)

The Organisational Identification Scale was used to investigate the level of identification with one’s organisation of affiliation. The OIS (Parker & Haridakis, 2008) is an 18-item scale measured on a 5-point Likert scale. The OIS is widely used in organisational and academic contexts to explore the relationship between identification, motivation, work engagement, and employee behaviour through its division into 4 factors: Management Connection (item example “*My colleagues and I frequently criticize management*”); Invested Self-Concept (item example “*I’d experience a sense of loss if I left the organization*”); Integrated Goals and Values (item example “*I share organization’s goals*”); and Coworker Connection (item example “*My coworkers help me make sense of what’s happening at work*”).

#### 3.2.2. The Inclusive Leadership Scale (ILS)

The Inclusive Leadership Scale was used to investigate the leadership style and, more specifically, the level of inclusive leadership within the organisation of affiliation. The ILS (Ashikali, 2019) is a 13-item scale based on a 5-point Likert scale. In this case as well, the scale has been frequently used in scientific research and is divided into two factors: Cognitive dimension (item example “*My supervisor encourages me to discuss diverse viewpoints and perspectives on problem-solving with colleagues*”) and Affective dimension (item example “*My supervisor stimulates me to actively participate in the team*”).

#### 3.2.3. Diversity Climate Questionnaire (DCQ)

The Diversity Climate Questionnaire is a scale used to assess the diversity climate within the organisation of affiliation. The DCQ, in its Italian validation (Paolillo et al., 2017), is a 9-item scale based on a 6-point Likert scale, divided into 3 factors: Organisational Fairness (3 items) assesses the fairness of management in implementing policies and procedures (item example “*Managers here have a track record of hiring and promoting employees objectively, regardless of their race, gender, sexual orientation, religion, or age.*”); Organisational Inclusion (3 items) examines the extent to which individuals from diverse backgrounds are structurally included or excluded (item example “*Managers here encourage the formation of employee network support groups*”); and Personal Diversity Value (3 items) reflects an individual’s perspective on the importance of diversity (item example “*I think that diverse viewpoints add value*”). In our study, we used only the fairness and inclusion factors, as indicated by the literature. We focused on the fairness and inclusion dimensions of Mor Barak’s diversity climate scale as these constructs are more directly aligned with the psychological mechanisms underlying the EVLN model. Specifically, fairness and inclusion are critical in shaping employees’ perceptions of organisational support and justice—key antecedents of constructive (Voice, Loyalty) and withdrawal (Neglect, Exit) behaviours.

#### 3.2.4. EVLN Model Questionnaire

The EVLN model (Exit, Voice, Loyalty, Neglect) measures employees’ response behaviours to dissatisfaction or stress in the workplace. It explores how individuals react when they are unhappy with their organisation or work conditions, with each dimension representing a different type of response.

Exit refers to the decision to leave the organisation, either by seeking a new job or quitting the position. This behaviour is a direct response to high dissatisfaction, where the employee feels that leaving is the only viable option. Voice, on the other hand, involves actively communicating problems or concerns within the organisation, aiming to find solutions that can improve the situation. Employees who adopt “voice” do not simply complain but actively attempt to change or improve the working conditions.

Loyalty represents a more passive response, where employees remain loyal to the organisation despite their dissatisfaction, without actively trying to change or improve things. They show trust and patience, expecting things to improve on their own. Lastly, Neglect refers to a passive disengagement behaviour, where employees reduce their effort and productivity, neglecting their responsibilities and contributing little to improving the situation. The EVLN model consists of 20 items, whose answers measure the degree of agreement on a 7-point Likert scale ranging from “Completely disagree” to “Completely agree” (item examples per factor: Exit “*Consider possibilities to change job*”; Voice “*Suggest solutions to your supervisor/manager*.”; Loyalty “*I am proud to say that I am working for this company.*”; Neglect “*Put less effort into your work than may be expected of you.*”). The scale was validated in 2008 and is used both in academic and organisational settings (Liljegren et al., 2008).

### 3.3. Data Analysis

The hypothesised model was tested using structural equation modelling (SEM) with AMOS version 27.0. The statistical analysis was carried out in two main phases: an exploratory phase, aimed at examining potential associations among the variables and assessing common method bias; and a confirmatory phase, focused on testing the hypothesised relationships. In the first phase, bivariate correlations were calculated among all variables included in the theoretical model (see Figure 1) using Pearson’s correlation coefficient (r), which measures the strength and direction of linear associations.

To assess potential common method bias, a confirmatory factor analysis (CFA) was performed, and model fit indices were evaluated. Additionally, the reliability and validity of the scales were assessed using Cronbach’s alpha and McDonald’s omega (Bonniga & Saraswathi, 2020).

In the second phase, a multiple mediation model was tested. Organisational Identification was treated as the independent variable, while Diversity Management and Inclusive Leadership were modelled as mediators. The four outcomes from the EVLN framework—Exit, Voice, Loyalty, and Neglect—were treated as dependent variables.

The mediation effects were examined simultaneously using the bias-corrected bootstrap method. A 95% confidence interval (CI) for the fully standardised indirect effects was computed based on 2000 bootstrap samples (Preacher & Hayes, 2008).

An indirect effect was considered statistically significant if both the lower and upper bounds of the CI were either positive or negative, indicating that the interval did not include zero. This criterion confirms the presence of a statistically significant mediation effect.

## 4. Results

### 4.1. Preliminary Analysis

#### 4.1.1. Test of Common Method Variance

Since the endogenous and exogenous variables were measured simultaneously and using the same instrument, we examined whether common method bias was present to ensure that these biases were not distorting the data. Considering the growing debate on the different approaches to test for common method bias, both the Harman single-factor test and the common latent factor method were applied. First, the Harman single-factor test involved performing an exploratory factor analysis with a single-factor, unrotated solution.

The findings indicated that the total variance explained by a single factor in the current sample was 15.06%, which is well below the recommended threshold of 50% (Podsakoff et al., 2003). This implies that common method bias is not present. Furthermore, a confirmatory factor analysis was conducted following Malhotra et al.’s (2006) recommendation that “*method bias is assumed to be substantial if the assumed model fits the data*”. The single-factor model tested demonstrated a poor fit with the data (CFI = 0.48; RMR = 0.315; RMSEA = 0.114), further confirming the absence of common method variance.

Lastly, using the common latent factor (CLF) method, the standardised regression weights of all items were compared between the models with and without the CLF. The differences in regression weights were minimal (Δ < 0.2), providing additional evidence that common method bias is not a significant concern in our data. The final model exhibited good fit indices: χ^2^(25) = 65.854, *p* < 0.001, χ^2^/df = 2.623, RMSEA = 0.07 (C.I. = 0.061–0.073), CFI = 0.94, GFI = 0.96, SRMR = 0.04.

#### 4.1.2. Intercorrelation Test Between All Variables

In Table 1, we can see the results of the correlations between all the variables, including the subscales under study, as well as the mean values and standard deviations. Specifically, the results show that almost all variables are significantly correlated with each other. The only variable that shows fewer significant links with the other variables in the EVLN model is the “exit” variable. However, it demonstrates an interesting correlation with management connection (r = 0.26, *p* < 0.001), suggesting that excessively control-oriented management may lead employees to consider leaving the organisation. Additionally, there is another noteworthy correlation with invested self-concept (r = −0.34, *p* < 0.001), indicating that when the organisation invests in the personal and professional growth of its employees, they are less likely to leave the organisation. All other variables subjected to correlation confirm the literature and theoretical connections that underpin our study, namely, that diversity management and inclusive leadership can have a significant impact on the relationship between organisational identification and employee satisfaction.

#### 4.1.3. Validity and Reliability

After verifying that there was no common method bias among the variables in our model, we deemed it appropriate to test the validity and reliability of the variables before proceeding with the mediation analysis. We calculated the Cronbach’s alpha, McDonald’s omega, Composite Reliability (CR), and Average Variance Extracted (AVE) for the variables included in the mediation: Inclusive Leadership, Organisational Identification, Exit, Voice, Loyalty, Neglect, and Diversity Climate. The results (see Table 2) show that all the variables considered demonstrate high levels of reliability and validity.

### 4.2. The Multiple Mediation Analysis

**Figure 2:**
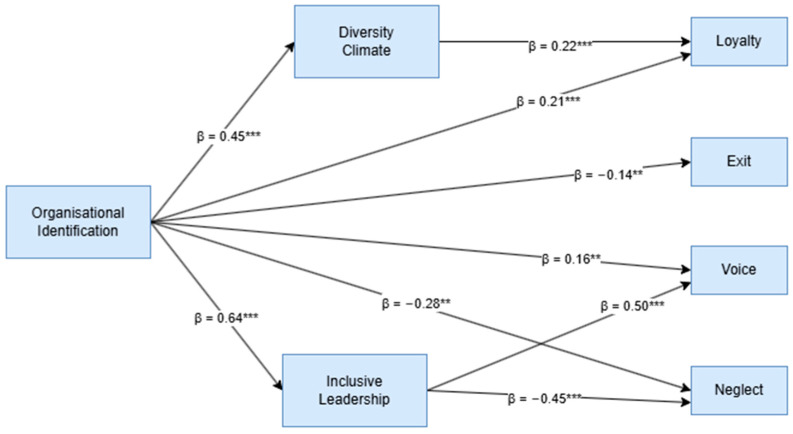
Structural model: *** *p* < 0.001, ** *p* < 0.05; non-significant links have not been reported.

After conducting the preliminary analyses, which supported our study, we proceeded with the mediation analysis following the model and hypotheses outlined in Figure 1. Mediation analysis is employed to examine the mechanism through which an independent variable influences a dependent variable via the inclusion of a third variable, known as the mediator. First, we examined the direct effect of the variables. Mediation analyses are commonly used to explore the mechanisms underlying complex relationships in empirical data (MacKinnon & Fairchild, 2009; Edwards & Lambert, 2007). In particular, mediation models are employed to investigate cases where the observed relationship between an independent variable, x, and a dependent variable, y, is clarified through a set of intermediate variables, m_1_, m_2_, ..., m_k_, referred to as mediators. In the context of the cause-and-effect relationship between x and y, mediators serve as intermediaries, where changes in x influence m_j_ (j = 1, ..., k), which in turn affect y. The x → m_j_ → y pathway illustrates the process by which x exerts either partial or complete effects on y (Baron & Kenny, 1986; Luo & Geng, 2016; Yuan et al., 2013). Figure 2 shows a visual representation of the results of a mediation model.

Specifically, the results reveal a significant direct effect of Organisational Identification on all four outcome variables: Exit (β = −0.14 **), Voice (β = 0.16 **), Loyalty (β = 0.21 **), and Neglect (β = −0.28 **). This indicates that Organisational Identification can significantly influence both employees’ positive responses toward the organisation and their negative responses. Therefore, Hypothesis H1 of our study is confirmed.

There is also a significant and positive direct effect of Organisational Identification on Diversity Climate (β = 0.45 **) and Inclusive Leadership (β = 0.64 **), highlighting the strong connection these three constructs share in relation to organisational practices. As a result, Hypotheses H2 and H3 of our study are also confirmed (see Figure 2).

Regarding the indirect effect of Diversity Climate and Inclusive Leadership on the relationship between Organisational Identification and both positive behavioural responses (Voice and Loyalty) and negative behavioural responses (Exit and Neglect), the results partially confirm Hypotheses H4 and H5. Specifically, Diversity Climate mediates the relationship between Organisational Identification and Loyalty [β = 0.05, *p* < 0.001, (C.I. 0.119–0.458)], revealing that a more inclusive climate promotes stronger identification among employees, leading to loyal behavioural outcomes. As for the mediating effect of Inclusive Leadership, it influences the relationship between Organisational Identification and Neglect [β = 0.05, *p* < 0.001, (C.I. 0.255–0.786)] and the relationship between Organisational Identification and Voice [β = 0.06, *p* < 0.001, (C.I. 0.278–0.987)]. These findings suggest that Inclusive Leadership plays a key role in fostering a Diversity Climate characterised by fairness and inclusion, which in turn enhances employees’ identification with the organisation. This strengthened identification, as suggested by Social Identity Theory, can increase employees’ engagement, participation, and behavioural focus on work-related processes (see Table 3).

## 5. Discussion and Conclusions

This study highlights the complex interplay between organisational identification (OID), diversity climate, inclusive leadership, and the EVLN (Exit, Voice, Loyalty, Neglect) model in shaping employee behaviour and related organisational outcomes. The findings indicate that diversity climate plays a pivotal role in fostering an inclusive work environment. A strong sense of organisational identification can shape how employees interpret their work environment, leading them to perceive the organisational climate as fair and socially integrative. This, in turn, promotes constructive behaviours (Voice, Loyalty) while reducing negative responses (Exit, Neglect).

The proposed model posits that organisational identification exerts a direct influence on job satisfaction, while inclusive leadership and diversity climate function as mediating variables that shape and strengthen this relationship. This conceptualisation highlights the complex interplay between individual identification processes and contextual organisational factors in determining employee attitudes.

The diversity climate acts as a contextual enabler, fostering a sense of value and support among employees. Inclusive leadership reinforces these effects by enhancing perceptions of fairness and psychological safety, thereby reducing the likelihood of disengagement in response to dissatisfaction.

These results underscore the theoretical significance of organisational identification as a key mechanism through which employees come to perceive and value diversity within the organisation, ultimately enhancing their engagement. Inclusive leadership further enhances this dynamic by ensuring the procedural justice and psychological safety necessary for meaningful employee participation in organisational life (Platania et al., 2022a, 2022b, 2022c). Leaders who embrace diversity and foster inclusion not only shape structural and cultural practices but also directly influence how employees perceive and engage with their work environment. This is consistent with the EVLN framework, whereby inclusive leadership mitigates withdrawal behaviours and fosters active, loyal engagement (Platania et al., 2022c).

These findings yield important theoretical implications: diversity and inclusion should not be viewed merely as normative or ethical imperatives but as foundational elements of effective organisational functioning.

A strong sense of organisational identification enhances employees’ perceptions of inclusion and belonging, which, in turn, buffers dissatisfaction and fosters engagement and loyalty. This integrative perspective bridges the gap between diversity management and behavioural models, demonstrating that embedding inclusive leadership within diversity strategies can amplify positive organisational outcomes (Platania et al., 2021; Buscemi et al., 2016).

Moreover, the study supports the multidimensional view of diversity climate, including intentionality, planning, and praxis, and points to the influential role of leadership in shaping how these dimensions are integrated into organisational practice.

In conclusion, this research advances our understanding of the psychological mechanisms linking inclusive leadership and organisational identification to employee behavioural responses in diverse workplace settings. The findings emphasise the importance of integrating models of inclusive leadership and employee behaviour to foster a more engaged and committed workforce. While the study does not explicitly examine the direct relationship between inclusive leadership and diversity climate, it lays a solid foundation for future research to explore these complex dynamics more deeply.

Limitations of the study and practical implicationsDespite the results obtained, the research presents some limitations that deserve consideration. Firstly, the sample used is predominantly composed of employees from the Italian public administration, which may limit the generalisability of the findings to other work contexts, such as the private sector or international organisations. It should also be noted that the effect of organisational identification on the behavioural responses outlined in the EVLN model may not be uniform across all organisational contexts. Specifically, the fact that the sample in the present study is composed solely of employees from public administration may have influenced the nature and intensity of the observed relationships.

In the public sector, organisational identification is often linked to values such as service, job stability, and social mission—factors that may encourage “voice” and “loyalty” responses even in the presence of dissatisfaction, thereby reducing the likelihood of “exit” compared to what might occur in private sector contexts, which are characterised by greater competitiveness, contractual flexibility, and profit orientation. Consequently, in organisations with different cultures and structures—such as private companies or multinational corporations—organisational identification might exert a different influence on employee behaviour.

Future research should therefore explore how these dynamics manifest in heterogeneous organisational contexts, to assess the generalisability of the findings and to understand whether, and to what extent, the link between organisational identification and EVLN responses is modulated by the type of organisation, institutional values, and the nature of the employment relationship.

Future studies could expand the sample by including different corporate realities to test the replicability of the results in different contexts.

Additionally, the measurement of diversity climate and inclusive leadership was based on self-report tools, which could be subject to social desirability bias. Future research could integrate qualitative methods, such as interviews or focus groups, to gain a more in-depth understanding of the organisational dynamics related to diversity and inclusion. Furthermore, the cross-sectional nature of the study limits the interpretability of the results, as the data were collected at a single point in time. A longitudinal study could provide more information and confirmation of the results obtained, focusing on the effectiveness of inclusion practices and leadership over time. Adopting a longitudinal approach would allow for the assessment of not only the immediate impact of diversity policies but also their sustainability and long-term effects on employee satisfaction and engagement.

Regarding practical implications, focusing on the intervention processes of inclusive leadership within organisational contexts means implementing concrete strategies aimed at fostering fairness, psychological safety, and participation among all employees. These processes may include training programmes for leaders to develop inclusive communication skills, feedback mechanisms that ensure all voices are heard, and decision-making practices that actively involve diverse team members. By embedding such inclusive practices, organisations not only enhance citizenship behaviours and managerial effectiveness but also generate positive social impact. This approach contributes to shifting from a traditional hierarchical model towards a circular structure based on shared and distributed responsibility.

## Figures and Tables

**Table 1:** Mean, standard deviation, and intercorrelations of variables.

		*M/SD*	1	2	3	4	5	6	7	8	9	10	11
**Diversity Climate**	Fairness (1)	3.9/1.1	−										
	Inclusion (2)	4.1/1.2	0.75 **	−									
**Inclusive Leadership**	Cognitive Dimension (3)	3.54/0.7	0.59 *	0.51 **	−								
	Affective Dimension (4)	3.65/0.9	0.56 **	0.52 **	0.87 **	−							
**Organisational Identification**	Management Connection (5)	2.98/0.7	−0.46 **	−0.45 **	−0.40 **	−0.50 **	−						
	Integrated Goal Values (6)	3.89/0.9	0.50 **	0.46 **	0.70 **	0.69 **	−52 **	−					
	Coworker Connection (7)	3.58/0.8	0.42 **	0.37 **	0.50 **	0.42 **	−0.38 **	0. 48 **	−				
	Invested Self-Concept (8)	3.21/0.9	0.29 **	0.17	0.46 **	0.45 **	−0.26 **	0.47 **	0.32 **	−			
**EVLN**	Exit (9)	2.98/0.6	−0.08	−0.13	−0.09	−0.18	0.26 **	−0.11	−0.04	−0.34 **	−		
	Voice (10)	4.98/0.8	0.38 **	0. 37 **	0.60 **	0.57 **	−0.34 **	0.50 **	0.40 **	0.28 **	−0.04	−	
	Loyalty (11)	3.75/1.	0.50 **	0.48 **	0.64 **	0.62 **	−0.60 **	0.66 **	0.46 **	0.31 **	−0.14	0.59 **	−
	Neglect (12)	2.66/0.9	−0.20 *	−0.26 **	−0.42 **	−0.41 **	0. 23 *	−0.34 **	−0.17	−25 **	0.22 *	−0.40 **	−0.26 **

** Correlations are significant at the *p* < 0.001 level; * Correlations are significant at the *p* < 0.05 level.

**Table 2:** Validity and reliability.

	α di Cronbach	ω di McDonald	AVE	CR
Inclusive Leadership	0.97	0.97	0.57	0.86
Organisational Identification	0.87	0.88	0.54	0.83
Exit	0.81	0.85	0.53	0.82
Voice	0.80	0.80	0.51	0.80
Loyalty	0.85	0.83	0.55	0.83
Neglect	0.91	0.91	0.55	0.84
Diversity Climate	0.82	0.82	0.56	0.88

**Table 3:** Standardised direct, indirect, and total effects of organisational identification on the EVLN model.

Outcome	Direct Effect (β)	Indirect Effect (β)	95% CI (LL–UL)	Total Effect (β)
Loyalty	0.21 **	0.05 ***	0.119–0.458	0.26
Neglect	−0.28 **	0.05 ***	0.255–0.786	−0.23
Voice	−0.16 *	0.06 ***	0.278–0.987	−0.10

Note: Indirect effects are mediated through Inclusive Leadership and Diversity Climate; total effects were computed by summing direct and indirect effects; * *p* < 0.05, ** *p* < 0.01, *** *p* < 0.001.

## Data Availability

Data for this study is available upon request. Participants were informed that any future use of their data would be by the current research team.
